# Supplementary material for: Highly nonlinear dipolar exciton-polaritons in bilayer MoS2
Source: Nat Commun. 2022 Oct 25;13:6341. doi: 10.1038/s41467-022-33940-3 (PMC9596727; doi:10.1038/s41467-022-33940-3)
Supplement: Supplementary file 1 — Supplementary Information [file 41467_2022_33940_MOESM1_ESM.pdf]

Supplementary Information

Highly nonlinear dipolar exciton-polaritons in bilayer MoS_2

Biswajit Datta[1,*], Mandeep Khatoniar[1,2], Prathmesh Deshmukh[1,2], Félix Thouin[3], Rezlind Bushati[1,2], Simone De Liberato[4], Stephane Kena Cohen[3], and Vinod M. Menon[1,2,*]

[1]*Department of Physics, City College of New York, New York, NY, USA*

[2]*Department of Physics, Graduate Center of the City University of New York (CUNY), New York, NY, USA*

[3]*Department of Engineering Physics, École Polytechnique de Montréal, Montréal, Quebec, Canada*

[4]*School of Physics and Astronomy, University of Southampton, Southampton, UK*

[*]bdatta@ccny.cuny.edu, vmenon@ccny.cuny.edu

I Device fabrication

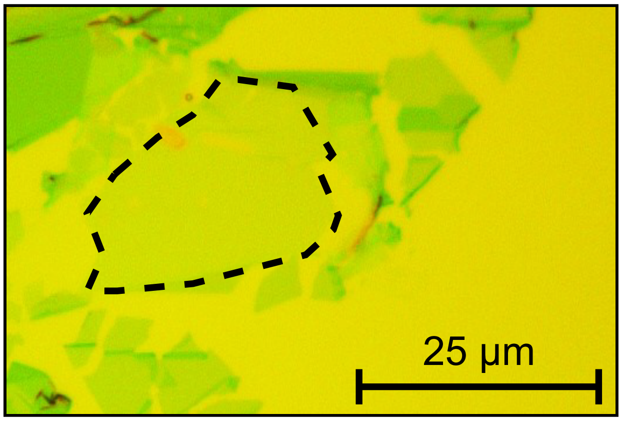

Fig. S1: Optical micrograph of the device. (a) Optical micrograph of the hBN/MoS_2 bilayer/hBN heterostructure on a distributed Bragg reflector (DBR) before top silver mirror deposition. The dashed line marks the uniform area of the heterostructure where measurements are done.

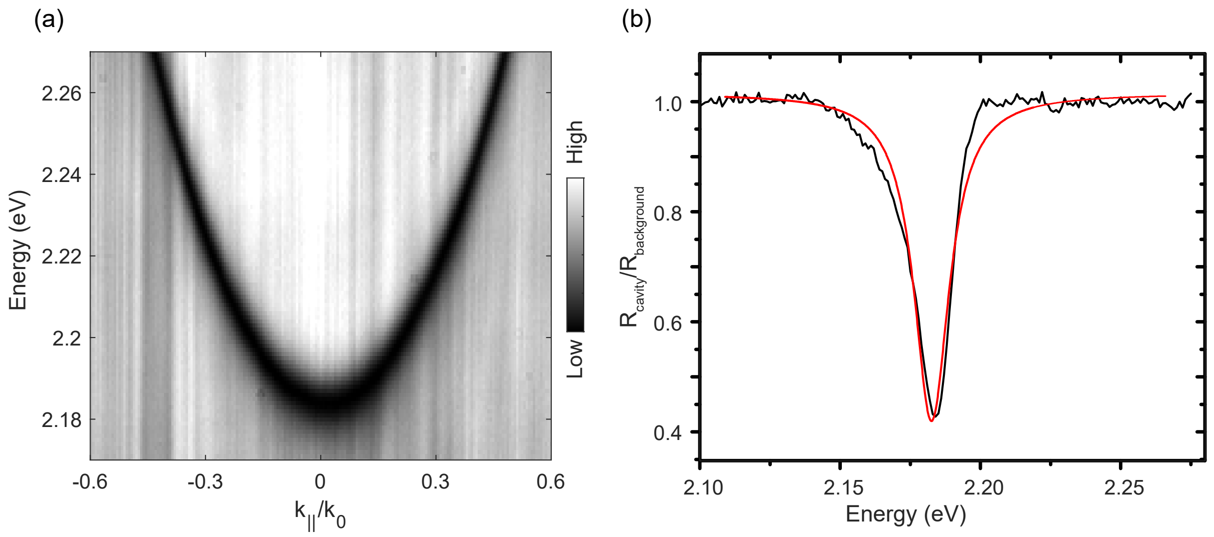

Fig. S2: PMMA bare cavity mode. (a) Dispersion of the PMMA bare cavity mode measured away from the hBN/bilayer MoS2/hBN heterostructure. The bare cavity mode gets 0.23 eV red shifted on the sample area due to the hBN and bilayer MoS_2. (b) Line cut of the bare cavity mode at $k_{||} = 0$. The red curve is the fitted Lorentzian with a FWHM of 15.5 meV.

II Details of coupled oscillator model

The five polariton branches in our experiment can be described with the eigen modes of the following five coupled oscillator model.

$$\begin{pmatrix} E_{cav} & \Omega_A/2 & \Omega_{IE}/2 & \Omega_{A2s}/2 & \Omega_B/2 \\ \Omega_A/2 & E_A & 0 & 0 & 0 \\ \Omega_{IE}/2 & 0 & E_{IL} & 0 & 0 \\ \Omega_{A2s}/2 & 0 & 0 & E_{A2s} & 0 \\ \Omega_B/2 & 0 & 0 & 0 & E_B \end{pmatrix}$$

where $E_{\mathrm{cav}} = \frac{c\hbar}{\mathrm{en_c}}\sqrt{\mathrm{k_v}^2 + k_{||}^2} = \frac{c\hbar}{\mathrm{en_c}}\sqrt{\mathrm{k_v}^2 + \left(10^7 \frac{k_{||}}{k_0}\right)^2}$, $k_v = \frac{2\pi}{\lambda_c} n_c$, $k_0 = \frac{2\pi}{\lambda_c} \approx 10^7\ \mathrm{nm}^{-1}$, e is the electronic charge. Here the wavelength is in meter and energy is in eV. The subscripts cav, A, IE, A2s, and B refer to the cavity mode, A exciton, imterlayer exciton, 2s state of A exciton, and B exciton respectively. Ω_X and E_X refer to the Rabi splitting and energy of the X exciton respectively.

The fitting yields $\lambda_c = 637.5 \pm 0.1$ nm, $n_c = 1.450 \pm 0.003$, $E_{A1s} = 1.9323 \pm 0.0003$ eV, $E_{IL} = 2.0014 \pm 0.0001$ eV, $E_{A2s} = 2.078 \pm 0.002$ eV, $E_B = 2.111 \pm 0.001$ eV and the Rabi splittings $\Omega_{A1s} = 40.4 \pm 0.3$ meV, $\Omega_{IL} = 21.4 \pm 0.1$ meV, $\Omega_{A2s} = 13 \pm 0.5$ meV, $\Omega_B = 51 \pm 0.4$ meV.

III Reflection measurements on bare exciton and exciton-polariton data at elevated temperature

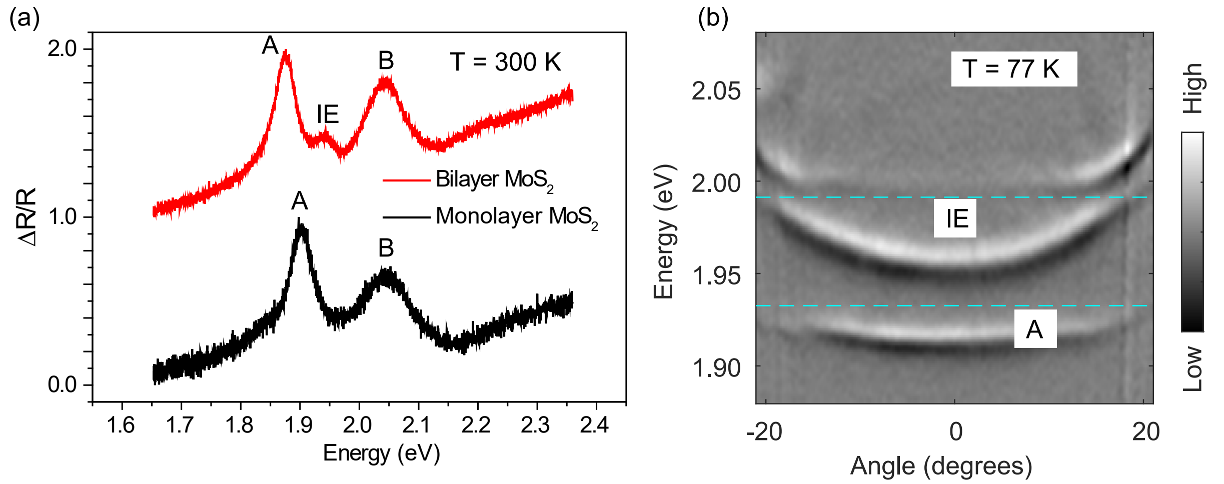

Fig. S3: White light reflection and exciton-polariton at elevated temperatures. (a) The red (black) color plot shows the differential reflection of bilayer (monolayer) MoS_2 on PDMS substrate. The small peak in between the A and B exciton in bilayer is the signature of the IE. (b) Strong coupling of the IE and A exciton at 77 K in bilayer MoS_2.

IV Details of the polariton density calculation

Polariton density is calculated from the Gross-Pitaevskii (GP) equation. The GP equation at wavevector k can be written as

$$i\hbar\frac{\partial\psi_{LP}(k,t)}{\partial t} = \left[\epsilon_{LP}(k) - \frac{i\hbar\gamma_{LP}}{2}\right]\psi_{LP}(k,t) + \hbar F_P(k,t) \tag{S1}$$

Here $\epsilon_{LP}(k) = \hbar\omega_{LP}(k)$ is the energy of the lower polariton of an exciton and γ_{LP} is the line width of the polariton. $F_P(k,t)$ is the coherent driving. The pulse width of our supercontinuum laser is 20 ps which is much larger than the polariton lifetime (30-50 fs). This allows the pumping to be treated as continuous wave which is resonant with our cavity. We can express the pumping term as $F_P(k,t) = F_P(k)e^{-i\omega_P t}$ and $\psi_{LP}(k,t) = \tilde{\psi}_{LP}(k)e^{-i\omega_P t}$ where ω_P is the pump frequency. This simplifies the above equation to

$$\left[\hbar\omega_P - \epsilon_{LP}(k) + \frac{i\hbar\gamma_{LP}}{2}\right]\tilde{\psi}_{LP}(k) = \hbar F_P(k) \tag{S2}$$

The $F_P(k)$ term can be written from the input-output relation.

$$F_P(k) = C(k)\sqrt{\frac{\eta \cdot P_{\text{int}}\ (k)}{\hbar\omega_P}} \tag{S3}$$

$$\eta = \frac{|t_{\text{top mirror}}|^2}{\tau_{\text{trip}}} \tag{S4}$$

Here C(k) is the photon Hopfield coefficient of the polariton branch. $P_{int}(k)$ is the incident power on the top mirror and η is the coupling coefficient. $t_{\text{top mirror}}$ is the transmission of top mirror, and τ_{trip} is the photon trip time in the cavity. This gives $\eta = 4.1 \times 10^{12}\ \text{s}^{-1}$. Equation S2 can be written as

$$\tilde{\psi}_{LP}(k, \omega_P) = \frac{\hbar F_P(k)}{\hbar\omega_P - \epsilon_{LP}(k) + \frac{i\hbar\gamma_{LP}}{2}} \tag{S5}$$

Polariton density at the wavevector k and frequency ω_P is

$$|\psi_{LP}(k, \omega_P, t)|^2 = \left|\tilde{\psi}_{LP}(k, \omega_P)\right|^2 = \frac{|C|^2 \frac{\eta \cdot P_{\text{int}}\ (k)}{\hbar\omega_P}}{(\omega_P - \omega_{LP}(k))^2 + \left(\frac{\gamma_{LP}}{2}\right)^2} \tag{S6}$$

Since the polaritons have a finite width in energy, the total polariton density at the wavevector k can be found out by integrating over the all frequency range

$$\left|\tilde{\psi}_{LP}(k)\right|^2 = \int \frac{|C|^2 \frac{\eta \cdot \xi(k, \omega_P)}{\hbar\omega_P}}{(\omega_P - \omega_{LP}(k))^2 + \left(\frac{\gamma_{LP}}{2}\right)^2} d\omega_P \tag{S7}$$

Here, $\xi(k, \omega_P)$ denotes the incident power density (power/frequency/wavevector).

The integral above can be numerically calculated but we can make an approximation to get a close form solution for the density. We note that the Lorentzian part of the integrand for $\gamma_{LP} \to 0$ is a Dirac delta function

$$\delta(\omega_P - \omega_{LP}) = \lim_{\gamma_{LP} \to 0} \frac{1}{\pi} \frac{\frac{\gamma_{LP}}{2}}{(\omega_P - \omega_{LP})^2 + (\frac{\gamma_{LP}}{2})^2} \tag{S8}$$

Using the above formula and taking the power density $\xi(k, \omega_P)$ constant over the narrow range

of excitation we get

$$\left|\tilde{\psi}_{LP}(k)\right|^2 \approx \frac{2\pi|C(k)|^2\eta\cdot\xi}{\hbar\omega_{LP}(k)\gamma_{LP}(k)} \tag{S9}$$

Here, $\gamma_{LP}(k)$ is determined from the experimental data.

Total real space polariton density can be found out by summing $\left|\tilde{\psi}_{LP}(k)\right|^2$ in the k-space for the experimental wavevector range

$$\begin{aligned}\left|\tilde{\psi}_{LP}\right|^2 &= \sum_{k=k_{min}}^{k_{max}} \left|\tilde{\psi}_{LP}(k)\right|^2 \\ &= \frac{2\pi\eta\cdot\xi}{\hbar}\sum_{k=k_{min}}^{k_{max}} \frac{|C(k)|^2}{\omega_{LP}(k)\gamma_{LP}(k)}\end{aligned} \tag{S10}$$

If P_0 is the measured real space peak power density (power/energy) of the pulsed supercontinuum laser excitation then $\xi = P_0/N$, where N is the number of k point in between k_{min} and k_{max}.

The above equation can also be written as

$$\left|\tilde{\psi}_{LP}\right|^2 = \sum_{k=k_{min}}^{k_{max}} \frac{2\pi}{\omega_{\text{LP}}(k)\,\tau_{\text{trip}}} \times \frac{|C(k)|^2|t_{\text{top mirror}}|^2\,R}{\gamma_{\text{LP}}(k)} \tag{S11}$$

Here, $\frac{2\pi}{\omega_{\text{LP}}\tau_{trip}} \sim 1$ and R is the incident number of photons/sec.

V Raw data for the nonlinearity of the IE polariton

Fig. S4a shows the differential reflection of the IE polaritons excited (with a 20 ps pulsewidth supercontinuum laser) in a narrow band used to study the nonlinearity of the IE polariton. Fig. S4b shows the differential reflection at the zero detuning $k_{\parallel}$ for IE polariton. The upper polariton branch red shifts and lower polariton branch blue shifts with increasing density reducing the Rabi splitting. We also notice that the two branches move asymmetrically – the lower branch moves more than the upper branch at zero detuning $k_{\parallel}$. This is because both exciton-exciton interaction and saturation give rise to blue shift for the lower branch but they produce opposite shift for the upper branch –

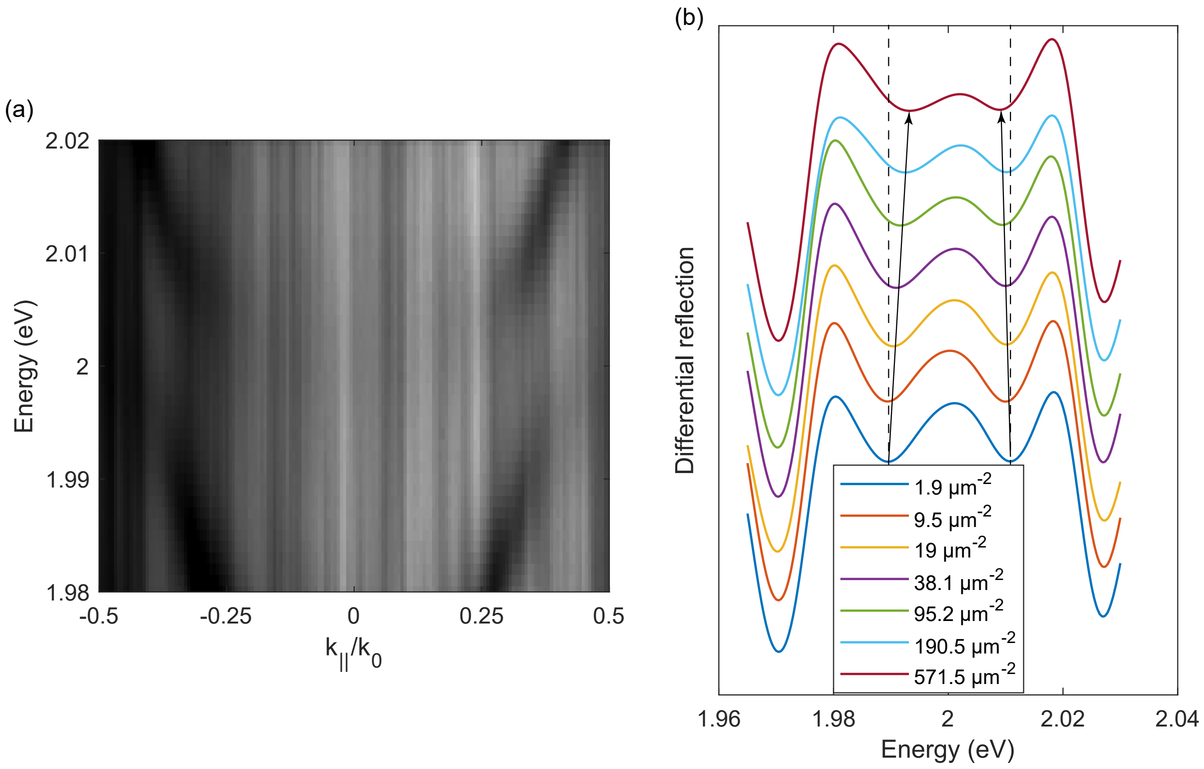

Fig. S4: Raw data for the nonlinearity of the IE polariton at 7 K. (a) K-space differential reflection data showing the dispersion of the IE lower branch (pol-2) and IE upper branch (pol-3) at the lowest density (1.9 µm^{-2}). The excitation bandwidth of the supercontinuum laser for the IE is set 40 meV by using a low-pass and a high-pass filter. (b) Line cut of the k-space data at zero detuning $k_{\|}$ showing the differential reflection of the IE lower branch (pol-2) and IE upper branch (pol-3) at different polariton density. Note that since the IE exciton blueshifts with increasing density, the zero detuning $k_{\|}$ also increases with increasing density. All the power dependent nonlinear measurements (for both samples in the cavity and outside the cavity) were carried out using a pulsed supercontinuum laser (20 ps pulsewidth) with proper bandpass filter in the input to excite only one polariton species.

as described in the main text.

VI Fits to determine the blueshift of the bare excitons

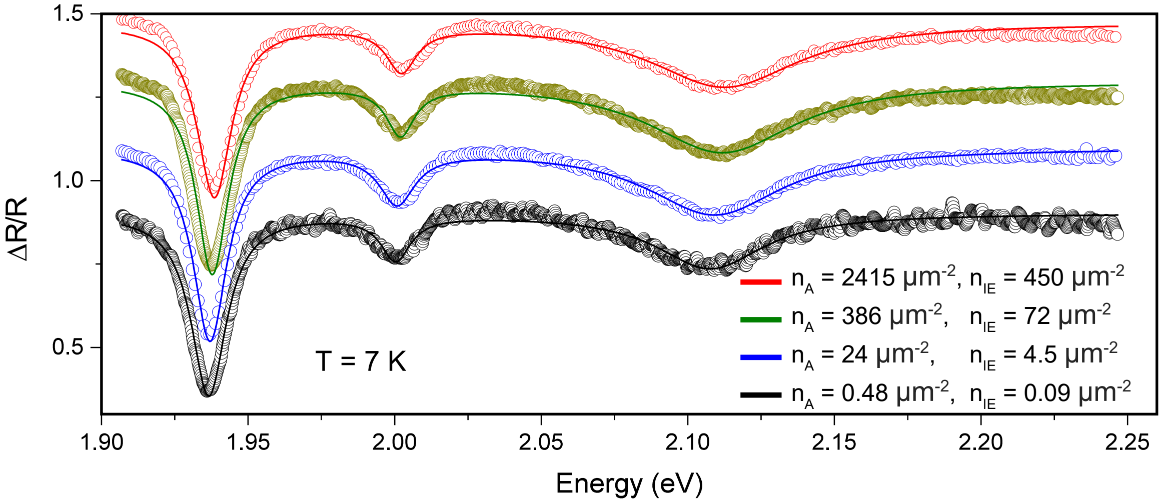

Fig. S5: Power dependent reflectance measurement to measure the blue shift. Three Lorentzian centered at A exciton, IE and B exciton respectively are fitted to the data at each power. The blue shift of the Lorentzians centered at A exciton and IE are plotted in the main text as a function of estimated exciton density. The method of exciton density estimation is described in Methods.

VII Rabi splitting of interlayer exciton polariton as a function of polariton density

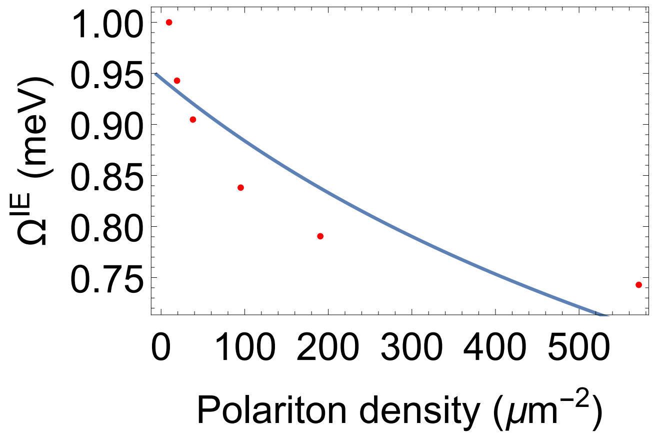

Fig. S6: Rabi splitting of IE polariton at the zero detuning $k_{\|}$ as a function of polariton density at 7 K. The blue line is the fit to the usual formula $\Omega = \frac{\Omega_0}{\sqrt{1+\frac{n}{n_{\mathrm{sat}}}}}$ for free 2D excitons.

We plot the Rabi splitting of the IE polariton at the zero detuning $k_{\|}$ as a function of density, see Fig. S6. We mote that the usual formula for free 2D excitons $\Omega = \frac{\Omega_0}{\sqrt{1+\frac{n}{n_{\mathrm{sat}}}}}$ does not fit our

data well. The inadequacy of the free 2D exciton formula to describe the density dependence of the Rabi splitting was also observed in a recent study[S1] on the Moiré exciton-polariton in a hetrobilayer TMDC.

VIII Energy of the lower branch of A exciton polariton as a function of polariton density

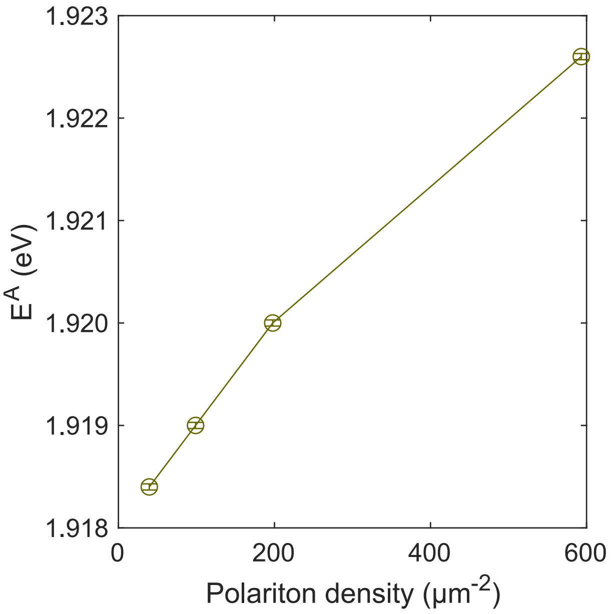

Fig. S7: Energy of the lower branch of A exciton polariton as a function of polariton density at 7 K from which the g_{LP}^{A} is calculated and plotted in Fig.3b of the main manuscript.

IX Separation of the saturation nonlinearity and exciton-exciton interaction nonlinearity for IE polariton

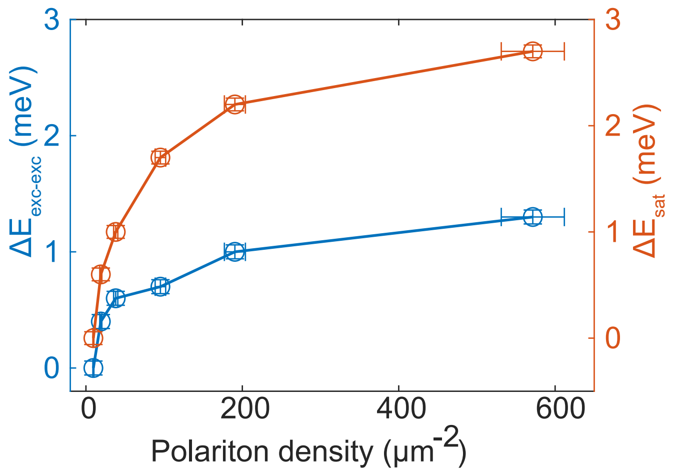

Fig. S8: Separation of the saturation nonlinearity and exciton-exciton interaction nonlinearity for IE polariton. Calculated $\Delta E_{exc-exc}$ and ΔE_{sat} at the zero detuning $k_{\|}$ from the experimental data as a function polariton density.

X Probing non-linearities in the reflectance spectrum of bilayer MoS_2 on two distinct time scales

Fig. S9a shows transient reflectivity measured on the bilayer film. The sample is first excited by an ultrashort pump pulse (200 fs) resonant with one of the exciton transitions. The relative change in reflectivity induced by the pump is then probed as a function of time delay using a broadband pulse. The transient reflectivity spectra obtained when pumping the A, B and IE resonances at a pump-probe delay of 1.5 ps are plotted in Fig. S9b. Regardless of the exciton transition that is initially excited, all the excitonic resonances redshift and saturate (which corresponds to the observed differential lineshapes). Time traces of the transient reflectivity are shown in Fig. S10.

In contrast, we notice that all the excitonic features blueshift when the sample is probed with a single broadband pulse lasting 20 ps (main text Fig. 1d). The opposite shifts observed using these two probes provide insight in the excitation regimes induced in the bilayers. When the probe is delayed from the pump all excitonic features are shifted to lower energies (Fig. S9a). This is consistent with the expected contribution of relaxed carriers in these materials[S2–S4]. Indeed, the pump-probe delay used in this experiment is much greater than the K-valley exciton lifetime (45 fs for A exciton and and 53 fs for IE). In this case, the probe interacts with the sample when only relaxed carriers remain.

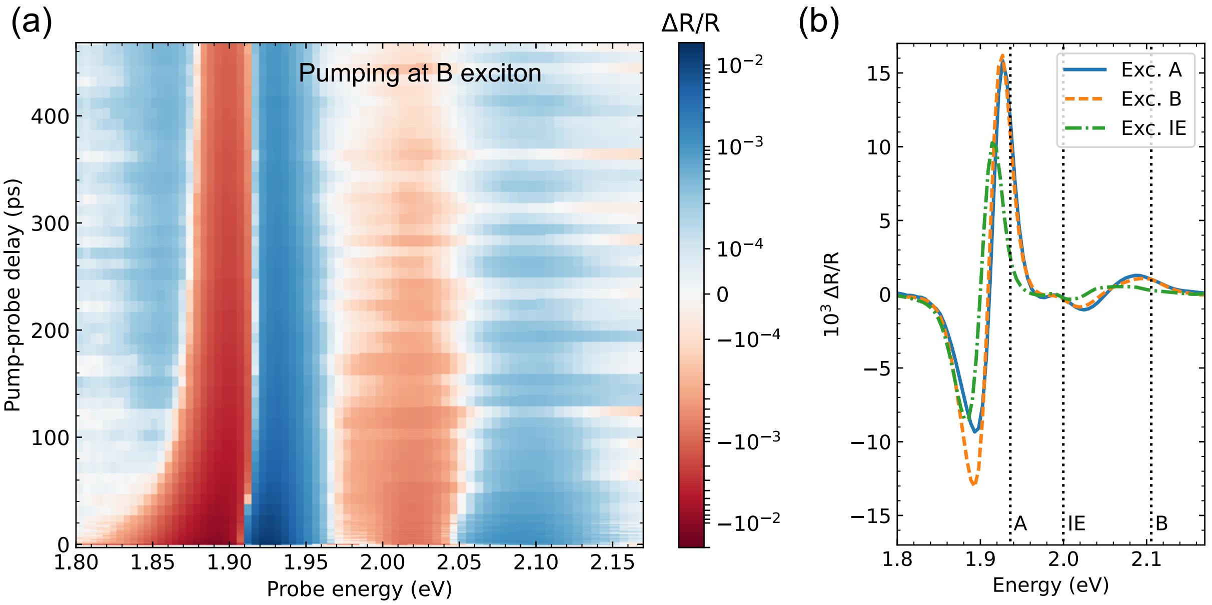

Fig. S9: Probing non-linearities in the reflectance spectrum of bilayer MoS_2 after the excitons scatter from K-valley. (a) Transient reflectivity spectra probed as a function of white-light probe energy and pump-probe delay. (b) Transient reflectivity spectra probed 1.5 ps after the resonant excitation of the A (full), B (dashed) and IE (dot-dashed) excitons. The absorbed photon density is about 4x10^4 photons/μm^2, 2x10^4 photons/μm^2 and 4x10^4 photons/μm^2 respectively.

When the reflectivity is probed while the material is still being pumped, as is the case in the single pulse reflectivity experiment, the dominant non-linear effect is instead a shift of all features to higher energies. Indeed, when the pulse interacts with the sample, A, B and IE excitons are continuously created while their constituents quickly relax out of the K valley. This quasi-continuous pumping leads to the steady-state presence of A, IE and B excitons amidst a growing background of relaxed carriers until the pulse no longer interacts with the sample. Moreover, the blueshift arising from excitonic repulsion dominates over the (smaller) redshift from the background of relaxed carriers. Given the long pulses used in our single pulse reflectivity, compared to the lifetime of the excitons ($\sim$ 50 fs), there are about 20 ps/50 fs = 400 times more relaxed carriers than excitons. The strength of the K-valley exciton-exciton interactions are therefore much higher than that due to the interaction with the background carriers.

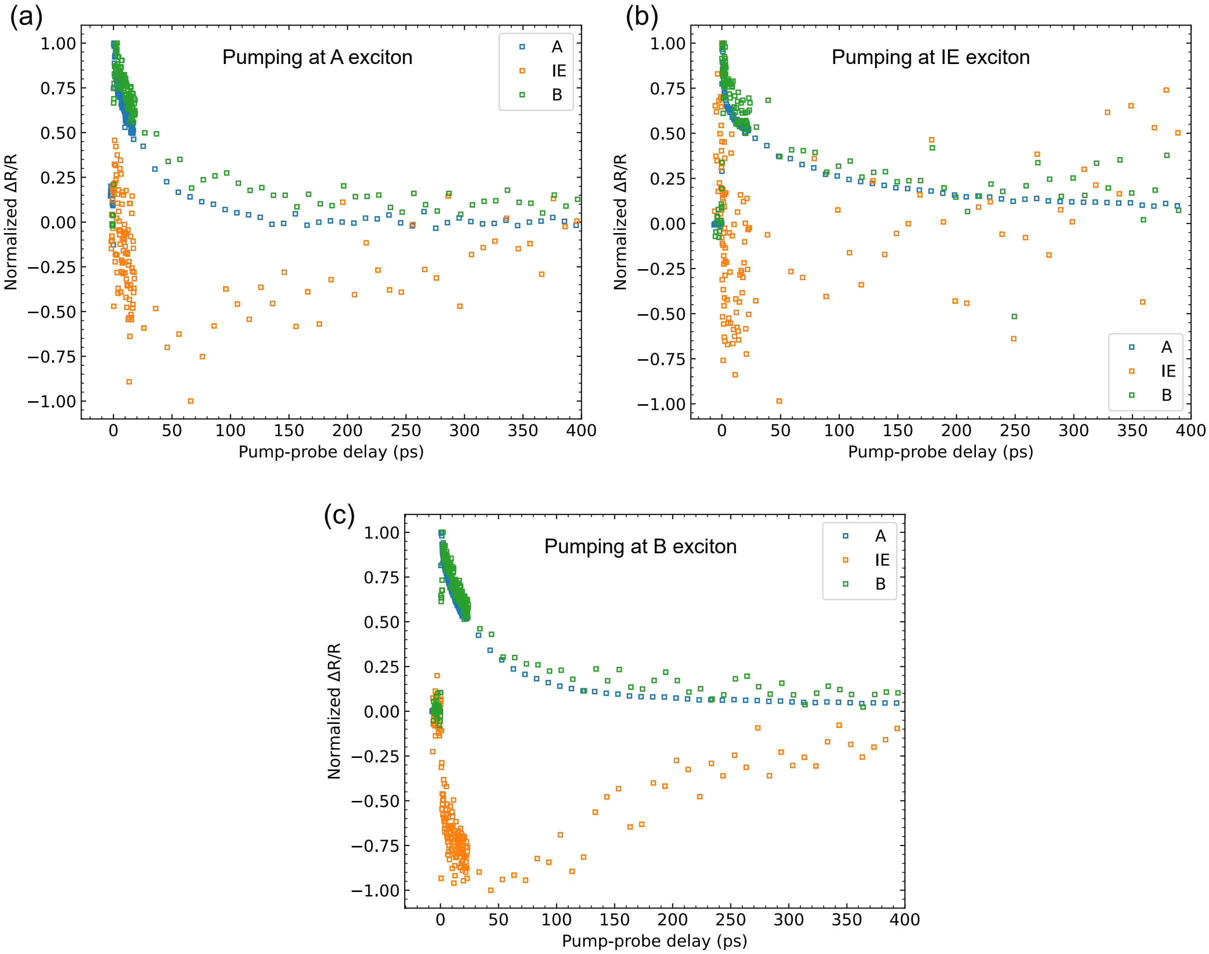

Fig. S10: Probing non-linearities in the reflectance spectrum of bilayer MoS_2 after the excitons scatter from K-valley. Transient reflectivity spectra probed as a function of white-light probe energy and pump-probe delay. The legend corresponds to the probe exciton energy where the time trace is taken.

References

[S1] Zhang, L. *et al.* Van der waals heterostructure polaritons with moiré-induced nonlinearity. *Nature* **591**, 61–65 (2021).

[S2] Schmidt, R. *et al.* Ultrafast coulomb-induced intervalley coupling in atomically thin ws2. *Nano letters* **16**, 2945–2950 (2016).

[S3] Steinhoff, A., Rosner, M., Jahnke, F., Wehling, T. O. & Gies, C. Influence of excited carriers on the optical and electronic properties of mos2. *Nano letters* **14**, 3743–3748 (2014).

[S4] Sie, E. J. *et al.* Observation of exciton redshift–blueshift crossover in monolayer ws2. *Nano letters* **17**, 4210–4216 (2017).
